# Biocompatibility Issues of Wound Dressings

**DOI:** 10.3390/bioengineering12111196

**Published:** 2025-11-02

**Authors:** Elga I. Alexander-Sinclair, Ekaterina S. Lapina, Nikita V. Edomenko, Denis V. Kostyakov, Evgeniy V. Zinoviev, Miralda I. Blinova, Natalia A. Mikhailova

**Affiliations:** 1 Institute of Cytology of the Russian Academy of Sciences, 194064 St. Petersburg, Russia; 2 I.I. Dzhanelidze Research Institute of Emergency Medicine, 192242 St. Petersburg, Russia

**Keywords:** bioengineered skin equivalents, biocompatibility, wound healing, wound dressings, The Dermal Equivalent, ED

## Abstract

This study examines the biocompatibility of 11 modern wound dressings (WDs)―Syspur-derm^®^, Parapran^®^, Lomatuell^®^H, Voskopran^®^, Metalline^®^, Granuflex^®^, Chitopran^®^, HydroTac^®^transparent, Branolind^®^N, Aquacel^TM^ adhesive foam, Aquacel^TM^Ag^+^―developed for the treatment of acute and chronic wounds, and their potential use as secondary WD for the hydrogel-based bioengineered skin equivalent (BSE) “Equivalent Dermal, ED”. The study was conducted to better understand the properties of these WDs that influence the healing process. The biocompatibility of WDs was evaluated in vitro based on their effects on the viability of human dermal fibroblasts (DFs). The MTT assay, lifetime analysis of DFs’ morphological state, and analysis of their actin cytoskeletal organization using a WDs’ extracts showed that effects of WD on DFs varied among WDs. It has been revealed that WDs Parapran^®^, Lomatuell^®^H, Voskopran^®^, Metalline^®^ and Chitopran^®^ have high biocompatibility and can be effectively used for wound treatment, whereas Granuflex^®^, Syspyr-derm^®^, HydroTac^®^ transparent, Branolind^®^N, Aquacel^TM^ adhesive foam and Aquacel^TM^Ag^+^ have lower biocompatibility, so they could be used for wound therapy with caution. Only Parapran^®^ with chlorhexidine showed high biocompatibility with the BSE “The Dermal Equivalent, ED” and can be safely used in combination with it as a secondary WD.

## 1. Introduction

Damage of the skin because of mechanical and thermal injuries, diabetes mellitus, genetic disorders, and surgical intervention leads to wounds and, as a result, to violation of skin functions, and primarily of the barrier–protective function [1]. Human body is equipped with natural mechanism of damage repair. Immediately after a wound occurs, the healing process begins with the goal of closing the wound as soon as possible. Healing is a dynamic process that can be divided into three stages: inflammatory, proliferative, and remodeling. These stages include four overlapping phases: hemostasis, inflammation, proliferation, and remodeling [1,2,3,4,5]. The healing time depends on several factors, including the degree of damage to the epidermis and dermis, the depth and shape of the wound, and its location. At any stage of healing, it can be negatively affected by both local factors (infection, hypothermia, radiation, oxygen burden in tissues, and pain) and systemic factors (the patient’s health, age, poor nutrition, and deficiency of proteins, vitamins, and minerals) [6,7]. This is one of the most vital, interactive, and complex processes occurring in the body. Whereas most small acute wounds do not require specialized treatment, larger and deeper wounds, both acute and chronic, need more qualified attention [8]. Currently, various strategic approaches are widely used for the treatment of acute and chronic wounds. There are large numbers of techniques to stimulate and accelerate the regeneration process. Most types of wound treatment are based on dressings. Various types of wound dressings are actively used. The components of these dressings can facilitate healing [9,10]. Wound dressing (WD) is a coating that is placed directly on the wound to protect it from further damage and to promote healing. The main functions of WD are to provide a moist environment for the wound, to maintain a normal temperature, to facilitate blood flow through the wound bed, to stimulate angiogenesis, granulation, and epithelialization, and to prevent bacterial infection. They also help to prevent further injury that could interfere with the natural healing process. The choice of the optimal WD is based on an understanding of its properties corresponding with the type, depth, location, and extent of the wound, as well as with the amount of discharge, infection, and adhesions. To achieve proper wound healing, it is crucial to have a true preference for particular dressing materials depending on the specific wound [6,8]. There is a dynamic interaction between WD and the wound environment. As wounds heal, the optimal type of WD can change depending on the changing wound healing environment, (such as its depth, amount of exudate, etc.) [11]. Moreover, successful selection WD depends on the knowledge of available dressings [12].

To date, many different dressings have been developed for various treatment protocols [13,14]. According to the time of their development, WDs can be divided into two categories: traditional (from 2000 BC to the 19th century) and modern (from the beginning of the 20th century to the present day). Traditional dressings typically include gauze, bandages, and plasters, as well as lint. In the past, plant leaves, honey, and spiderwebs were also used to help prevent bleeding. These traditional dressings formed the foundation for the creation of modern WDs. Advances in materials, in science, in nano- and biotechnology have led to the development of numerous modern WDs in various forms with different physical, chemical, and biological properties based on the various types of wounds [14,15,16,17]. However, there is no generally accepted classification of WDs. The factors listed below are related to WD-classification. Based on the type of contact with the wound, WDs can be divided into two categories: primary (direct contact with the wound) and secondary (applied on the top of primary WD to hold them in place on the wound). In accordance with to the origin of the WDs material, they are divided into three categories: plant, animal, and synthetic. WDs are available in various forms such as cloth (woven and non-woven), meshes, films, membranes, sponges, foams, hydrocolloids, hydrogels, ointments, adhesives, and combinations of these forms [6]. Depending on the interaction between WD and biological tissue, two types of WDs can be distinguished: passive and active. The simplest form of passive WDs is a sterile gauze that covers the wound surface and absorbs any secretions [15]. Active WDs, in turn, depending on their properties, can be divided into three categories: interactive (semi-permeable films and foams), advanced interactive (polymer, nanofiber, hydrogel, and hydrocolloid WDs), and bioactive WDs [11]. Interactive WDs create an occlusive environment that provide moisture and reduces the risk of bacterial infections. Bioactive WDs represent the most advanced group of wound care products, which include WDs containing drugs or biological factors. The use of cell-based technologies in the development of dressings has led to the creation of bioactive WDs that promote wound healing by normalizing impaired intercellular communication and reducing persistent inflammation. Among them, is devoted to bioengineered skin equivalents (BSEs) of various levels of complexity (epidermal, dermal, dermo/epidermal) based on natural or synthetic biopolymers. These skin equivalents can be found in different forms, such as films, hydrogels, and nanofibers. Some of these skin equivalents may also contain a cellular component, while others do not. The advantage of bioactive WDs is its ability to deliver biological molecules, such as growth factors, cytokines, and extracellular matrix components, as well as cells like fibroblasts, keratinocytes, melanocytes, and macrophages, which are necessary for activating regeneration processes [16,17,18]. BSE are recommended for the treatment of various types of wounds, including severe burns, deep wounds with tissue loss, uninfected diabetic neuropathic ulcers (full thickness), venous ulcers, and diabetic foot ulcers. Well-known examples of commercially available BSE are dermal BSE Dermagraft^®^ (Organogenic, Inc., Canton, MA, USA), dermo/epidermal BSE Apligraf^®^ (Organogenic, Inc., Canton, MA, USA), Theraskin^®^ (Soluble Systems LLC, Newport News, VA, USA), OrCel^®^ (Forticell Bioscience, Inc., New York City, NY, USA) and StrataGraft^®^ (Mallinckrodt Pharmaceuticals, Louis, MO, USA), epidermal BSE Laserskin^®^ (Fidia Advanced Polymeric, Abano Terme, Italy) and MySkin^®^ (Celltran Ltd., Sheffield, UK). [19]. One of the first BSEs developed in Russia is “The Dermal Equivalent, ED”, which is made from a type I collagen-based hydrogel that includes dermal fibroblasts (DF) isolated from the dermis of human skin as a cellular component. Collagen hydrogels have become attractive to WDs due to their low immunogenicity and unique abilities to accelerate cell proliferation, vascularization, and the development of granulation tissue. These features make them useful for reducing wounds and preventing scar formation [20].

Numerous clinical studies have demonstrated the effectiveness of this dermal equivalent in healing all types of skin wounds [21,22]. At the same time, clinicians have always wondered about choosing a suitable secondary WD that can help fix the BSE on a wound, protect it from drying out and environmental factors, and damage caused by dressings. Hydrogels, in general, are a promising type of wound dressing due to their unique structure, biodegradability, ability to incorporate biological molecules, and controlled release of therapeutic agents. They can effectively and easily combine different types of therapeutic cells [23,24]. However, in most cases, hydrogel WDs require the application of a biocompatible secondary WD to ensure adhesion to the wound bed and prevent adherence to the dressing material and drying out [11]. The choice of such a secondary WD is often based on experience and may not always be the best option.

Despite the wide range of WDs developed in recent years, the treatment of chronic wounds remains an extremely difficult and challenging task. The poor effectiveness of the available WDs can be explained by several related problems, including the impossibility of creating a universal WD, as well as the existing gap between the results of research on new WDs and commercially available products [25,26]. At the same time, it should be noted that despite such a wide variety of available WDs, there is still no comprehensive study of the benefits of one WD over the other, as well as of the potential for their combined application to optimize wound healing. The recent emergence of new, modern WDs developed by various manufacturers, which come in different types and with varying characteristics, deepens our current misunderstanding. The diversity of existing WDs and the absence of standardized protocols may limit their clinical utility or make them ineffective [10,17,18]. Thus, despite the positive clinical data on the effectiveness of BSE «The Dermal Equivalent, ED”, its use is limited. Research is ongoing to find a secondary WD for it to create synergies and optimal conditions for maintaining the viability and effectiveness of its cellular component, fibroblasts. The aim of this study was to compare the in vitro biocompatibility of 11 commercial WDs, as well as their potential use as secondary WD for BSE (“The Dermal Equivalent”, ED). All experimental procedures followed the International Organization for Standardization (ISO) 10993-5 [27]. The choice of these WDs is justified by their inclusion in the list of medical products recommended by national clinical guidelines [28] and the procedure for providing medical care [29] according to the profile “surgery (combustiology)”.

## 2. Results and Discussion

### 2.1. In Vitro Biocompatibility of WDs with Cultured DFs (2D-Model)

**Figure 1:**
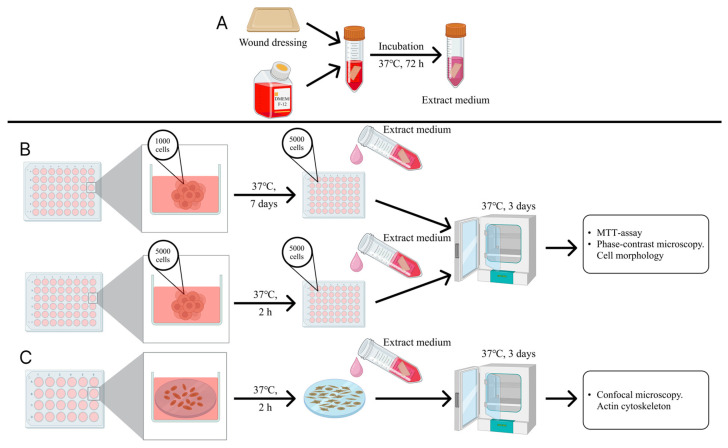
Schematic representation of the in vitro biocompatibility investigation of wound coatings (2D-model). Preparing liquid extracts from WD (**A**), analysis of the effect of WS extracts on the metabolic activity of human DFs (**B**) and the organization of their actin cytoskeleton (**C**).

Modern WDs developed into priority choice for treating various types of wounds, as they play a significant role in creating a favorable environment for healing and protecting wounds from infection and further damage [25,26]. Meanwhile, biocompatibility is a deciding factor, when selecting any medical material for use at the patient’s bedside. It is also essential that the material used in the regeneration process is no cytotoxic to healthy cells in the regenerating tissue [30]. To evaluate the biocompatibility of WDs, we investigated the effect of WDs’ extracts (Figure 1A) on the viability of human DF cultured in these extracts. First, standard culture medium was replaced with extracts immediately after cell adhesion and 7 days after their adhesion, the cell morphology was analyzed during the cultivation process, and an MTT assay was performed after 3 days of cell culture in these extracts (Figure 1B). The next step of the study was to investigate the effect of WDs’ extracts on the organization of the DFs actin cytoskeleton (Figure 1C).

**Figure 2:**
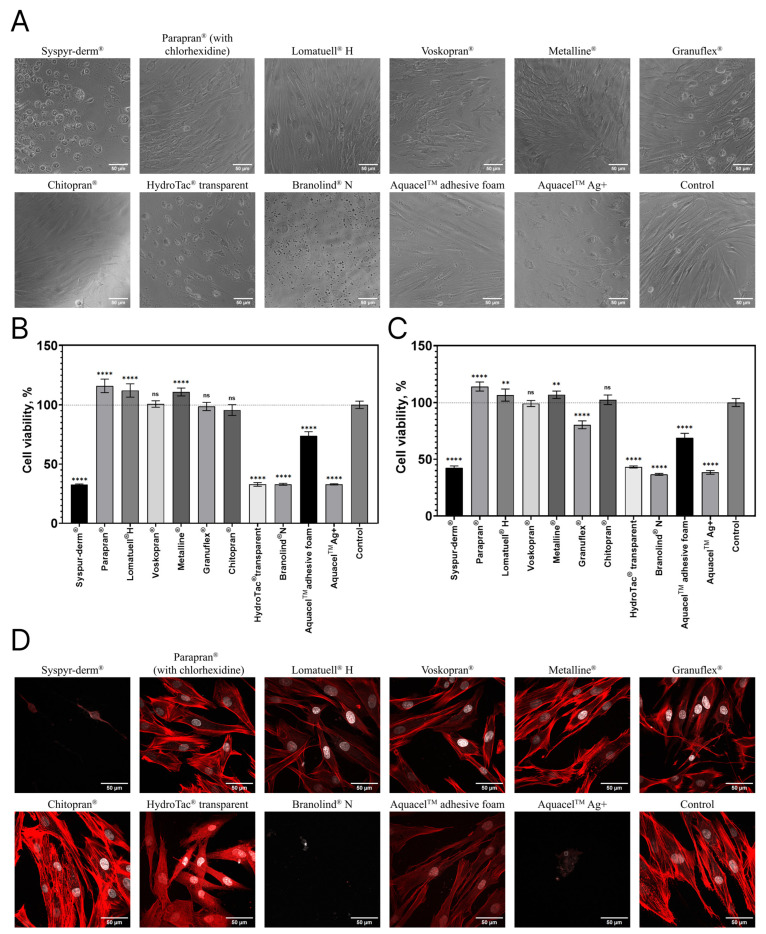
In vitro biocompatibility of wound dressings (WDs) with human DF cells cultured for 3 days in WD’s extracts (2D model). Image of light microscopy showing cell morphology (**A**). MTT assay of DFs viability cultured in extracts added immediately after cell adhesion (**B**) and 7 days after adhesion (**C**). Fluorescence images showing organization of the actin cytoskeleton (**D**). One-way ANOVA, Tukey’s HSD test: ****—*p* < 0.0001, **—*p* < 0.01, ns—not significant, compared to control.

It was found that the extracts differ in their effect on cultured human DFs (Figure 2).

The results of the MTT assay (Figure 2B,C) show that the viability of cells cultured in media based on extracts from Voskopran^®^ and Chitopran^®^ was similar to the control in both experiments. The extract from Granuflex^®^, however, had a slight cytotoxic effect in the second experiment, while in the first experiment, cell viability was similar to that of the control. The viability of DFs cultured in extracts from Parapran^®^, Lomatuell^®^ H, and Metalline^®^ in both experiments was higher than in the control group. The cytotoxic effect of the extract from Aquacel^TM^ adhesive foam on human DFs was moderate. However, Syspur-derm^®^, HydroTac^®^ transparent, Branolind^®^ N and Aquacel^TM^Ag^+^ had a pronounced cytotoxic effect on human DFs in culture. The results of the MTT assay are generally consistent with those of the analysis of cell morphology (Figure 2A). As can be seen from the images, DFs in the control group are well-spread with a typical morphology, forming a confluent monolayer. The monolayer organization and the morphological state of the cells cultured in extracts from Voskopran^®^ and Chitopran^®^ are comparable to those in the control group. The monolayer formed by the cells cultured in extracts from Parapran^®^, Lomatuell^®^ H and Metalline^®^ appears to be visually denser compared to the control. The latter is in good agreement with the results of the MTT assay and may be related to an increase in the proliferation activity of DFs. The cells cultured in the extracts from Granuflex^®^ and Aquacel™ adhesive foam form a monolayer, but their morphology differs from the control group. The cell structure is granular and there are numerous artifacts in the culture medium surrounding them. At the same time, when DFs are cultured in a Granuflex^®^ extract, some of the cells become rounded and float, and the morphological state of the monolayer indicates the beginning of destruction processes: the cells thin out and lose intercellular connections. Monolayer degradation is even more pronounced when DFs are cultured in extracts from Syspyr-derm^®^, HydroTac^®^ transparent, Branolind^®^ N, and Aquacel^TM^Ag^+^. At the same time, complete destruction of the monolayer was observed in the Syspyr-derm^®^ extract: almost all cells were rounded, swollen, and floating in a medium with a large number of artifacts. Considering that fibroblasts respond to chemotactic signals during injury, which stimulate not only their proliferation but also their migration to the affected area, mechanical interactions between cells and the extracellular environment depend on their mechanical rigidity, provided by the cytoskeleton. In such mechanical events as cell mobility or the establishment of cell polarity, continuous rearrangement of the actin filaments occurs through their polymerization and depolymerization [26,27]. The next step of the study was to investigate the effect of WDs’ extracts on the organization of the DFs actin cytoskeleton. It was revealed (Figure 2D) that in the control group, DFs were well-spread and had a shape and size characteristic of this cell type. A well-organized actin cytoskeleton was uniformly distributed throughout the cell. Pronounced stress fibers and focal adhesions were observed, with no actin aggregates being detected, and the nuclei had clear boundaries. In extracts from Parapran^®^, Lomatuell^®^ H, Voskopran^®^, Metalline^®^, and Chitopran^®^, the organization of the actin cytoskeleton was comparable to that of the control group. In the Granuflex^®^ extract, the overall organization of the actin cytoskeleton was similar to control group, but the cells appeared to be less spread and sometimes thinner. Stress fibers were not well defined, and the diffuse areas of increased actin fluorescence were observed. In the extract based on Aquacel^TM^ adhesive foam, the cells were well spread, but stress fibers and focal adhesions were not clearly defined, with only thin strands of actin filaments being detected at cell periphery. In the extract based on HydroTac^®^ transparent, the cells were less spread compared to the control; stress fibers and focal adhesions were poorly defined, and the actin cytoskeleton appeared as diffuse actin aggregates evenly distributed throughout the cytoplasm. In extracts from Syspyr-derm^®^, Branolind^®^ N, and Aquacel^TM^Ag^+^, only single round-shaped cells were identified, in which the organization of the actin cytoskeleton was practically not observed, which may indicate the destruction of the actin filaments.

The results of the analysis of the organization of the actin cytoskeleton were consistent with the results of the MTT assay and the lifetime analysis of the morphological state of the cells cultured in WDs’ extracts (Figure 2). Generally, based on these results, we can conclude that the particular WDs studied had different effects on the morpho-functional properties of cultured DFs and, consequently, and according to the degree of their biocompatibility (Table 1).

Considering that DFs are the main cellular protagonists of wound healing, and the healing process is associated with their ability to perform functions like proliferation, extracellular matrix secretion, and migration, it is crucial to understand the influence of WDs on the functional state of DFs when choosing a WD for wound therapy. Our in vitro study of the effect of various modern WDs on the viability of human DFs in a 2D model showed that Parapran^®^, Lomatuell^®^ H, Voskopran^®^, Metalline^®^, Chitopran^®^ have high biocompatibility and can effectively be used in the treatment of acute and chronic wounds. Despite the fact that WDs Granuflex^®^, Syspyr-derm^®^, HydroTac^®^ transparent, Branolind^®^ N, Aquacel^TM^ adhesive foam and Aquacel^TM^Ag^+^ have been claimed to accelerate regenerative processes and are recommended for treating chronic wounds of various origins (Table 2), choosing them for wound therapy should be based on the specific therapeutic properties of each WD. Moreover, potential negative impact of these WDs on the functional status of the DFs should also be taken into consideration.

### 2.2. In Vitro Biocompatibility of WDs with BSE “The Dermal Equivalent, ED” (3D Model)

**Figure 3:**
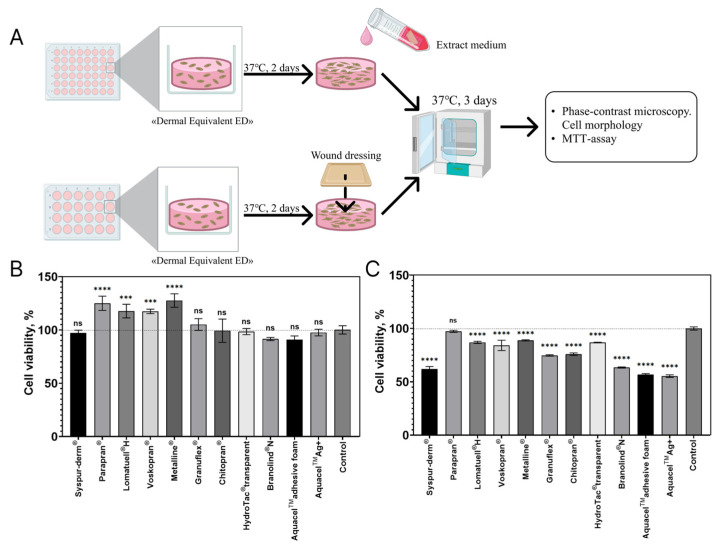
In vitro biocompatibility of wound dressings (WDs) with BCE “The Dermal Equivalent, ED” (3D model). Schematic illustration of the in vitro study design (**A**). MTT assay of the viability of DFs inside CH after incubation for 3 days with WD’s extracts (**B**) and in direct contact with WDs (**C**). One-way ANOVA, Tukey’s HSD test: ****—*p* < 0.0001,***—*p* < 0.001, ns—not significant, compared to control.

Due to its structure and composition, BSE “The Dermal Equivalent, ED” is a promising type of WD for accelerating regeneration. However, this WD requires secondary WD, which achieves synergy and optimal conditions for maintaining the viability and effectiveness of the cellular component (fibroblasts) within it. To evaluate the potential use of 11 different types of modern commercial WDs as secondary WDs for the BSE “The Dermal Equivalent, ED”, we investigated the effect of WD data on the viability of human DFs cultured inside collagen hydrogel (CH) as part of the BSE “The Dermal Equivalent, ED” using the MTT assay, which we adapted for 3D conditions. The effect of WD on the metabolic activity of human DFs cultured inside a CH, as part of a BSE “The Dermal Equivalent, ED” was evaluated using two methods: the extraction method and the direct contact method (Figure 3A). In the first set of experiments, we used the extraction method by replacing the standard culture medium around CH containing DFs with extracts based on WDs, two days after the polymerization of CH. Considering the inevitable contact between BSE “The Dermal Equivalent, ED” and the studied WDs when used as a secondary WD, a model was created to evaluate their biocompatibility through direct contact in vitro. WDs’ fragments were placed on the surface of the BSE “The Dermal Equivalent, ED”. After 3 days of co-incubation of BSE “The Dermal Equivalent, ED” with WDs or their extracts, the viability of DFs in the CH of the BSE was assessed using the MTT assay.

The results of the MTT assay demonstrated that WDs’ extracts did not have cytotoxic effects on DFs cultured in CH. However, they differed in their impact on the metabolic activity of DFs (Figure 3B). The viability of DFs in CH in the presence of extracts from Parapran^®^, Lomatuell^®^ H, Voskopran^®^ and Metalline^®^ was higher than in the control group, which is also in line with the findings of the study on the effect of these mediums on DFs under 2D culture conditions (Figure 2). The viability of DFs in CH in the presence of extracts from Syspyr-derm^®^, Granuflex^®^, Chitopran^®^, HydroTac^®^ transparent, Branolind^®^ N, and Aquacel^TM^ adhesive foam, Aquacel^TM^Ag^+^ is comparable to that of the control.

It was found that when WDs were in direct contact with BSE “The Dermal Equivalent, ED”, only Parapran^®^ had a high level of biocompatibility, while the other WDs had a cytotoxic effect on the DFs inside the CH (Figure 3C, Table 3).

### 2.3. In Vitro 2D and 3D Models of WD Biocompatibility as a Basis for Rational Choice

In vitro cytotoxicity assays are commonly used as a first step to ensure the biocompatibility of various medical devices. The WDs tested in this study are designed to treat acute and chronic wounds and are recommended for use according to national clinical guidelines in the field of surgery (combustiology). They belong to different categories of WDs and have various optimal characteristics for wound care, including safety, atraumatism, preservation of a suitable moist environment, and control of exudates (Table 2). The presented range of dressing materials enables the selection of a WD that is appropriate for the stage of the wound healing process.

In vitro biocompatibility tests, conducted according to the internationally established standards ISO 10993-5 [27], are crucial for predicting and simulating biological reactions to materials when placed in or on tissues. Any failure of in vitro cytotoxicity should be investigated to determine the risk of in vivo toxicity as it is a cause for concern. Each instance of in vitro cytotoxicity failure should be examined to ascertain whether there is a risk, although this does not suggest that a WD is not biocompatible in all cases. The use of various model cellular systems, ranging from simple 2D models to more complex 3D constructs, with different cell types such as fibroblasts, keratinocytes, and endotheliocytes, can help to optimize the selection of WD by taking into account the specific stage of wound healing. Additionally, the use of autologous cellular materials in these models can contribute in choosing the most suitable WD, taking into account individual intolerance to its components. This multi-level approach could contribute to selecting the optimal WD and improving the overall effectiveness of the wound treatment strategy.

The results of this study show that the tested WDs vary in their degree of biocompatibility with DFs and the BSE “The Dermal Equivalent, ED” (Table 4), and therefore, suggest the possibility of using them in clinic.

The results of in vitro biocompatibility studies of WDs with DFs (2D model) using WDs’ extracts showed that Parapran^®^, Lomatuell^®^ H, Voskopran^®^, Metalline^®^, Chitopran^®^ have high biocompatibility. However, extracts of Syspyr-derm^®^, HydroTac^®^ transparent, Branolind^®^ N, and Aquacel^TM^ adhesive foam and Aquacel^TM^ Ag demonstrated high cytotoxicity towards DFs in the 2D model. The results of an in vitro biocompatibility study of WDs with BSE “The Dermal Equivalent, ED” (3D model) using extracts indicate their sufficiently high biocompatibility with DFs cultured in hydrogels. In this case, BSE “The Dermal Equivalent, ED” can be considered as an experimental model of the dermis of the skin, rather than WD. Based on the results of the in vitro biocompatibility studies of WDs with cultured DFs (2D and 3D models), it can be assumed that the following WDs can be safely used for the treatment of acute and chronic wounds: WDs Parapran^®^, Lomatuell^®^H, Voskopran^®^, Metalline^®^, and Chitopran^®^. However, the choice of WD, such as Granuflex^®^, Syspyr-derm^®^, HydroTac^®^ transparent, Branolind^®^ N, Aquacel^TM^ adhesive foam and Aquacel^TM^ Ag^+^, should be made based on specific therapeutic properties of each WD, and by taking into account their possible negative impact on DF functions.

To assess the potential use of WD as a secondary coating for BSE “The Dermal Equivalent, ED”, a direct contact method is essential. Our studies showed that only Parapran^®^ with chlorhexidine demonstrated high biocompatibility with BSE “The Dermal Equivalent, ED”. Even though the results of studies with extracts from Lomatuell^®^ H, Voskopran^®^ and Metalline^®^ have demonstrated high biocompatibility of these WDs with DFs cultured both under 2D conditions and within the CH as part of the BSE, these WDs showed a slight cytotoxic effect, when placed in direct contact with the hydrogel BSE (Table 4). This may be due to the sorbing properties of these WDs as claimed by the manufacturer (Table 2), which can negatively affect the moisture content of the BSE and, consequently, the viability of the cells in it. These WDs can be used as secondary WD to the BSE “The Dermal Equivalent, ED”, but with extreme caution and regular monitoring of the wound’s condition. The use of HydroTac^®^ transparent for this purpose requires even greater caution, despite the fact that this WD demonstrated a similar, insignificant cytotoxic effect, when placed in direct contact with BSE, and extract based on HydroTac^®^ transparent under 3-D conditions, did not have any cytotoxic effects at all. However, when tested under 2-D conditions, an extract based on this product had a strong cytotoxic effect on cultured DFs, which may be due to the presence of propylene glycol in the WD. Propylene glycol, also known as propane-1,2-diol (PD), is a widely used ingredient in the pharmaceutical industry for creating various medicines and medical products. This is because PD has the ability to act as a cryoprotectant and solvent for different active substances, allowing creation of stable and effective medication formulations. However, clinical studies have reported serious side effects, including the development of renal failure in patients receiving PD as a drug carrier. Data from in vitro studies using various cellular test systems confirm the cytotoxicity of PD against cultured cells and are thus consistent with the results of our studies [36,37,38]. The lack of cytotoxic effect when testing the HydroTac^®^ extract in a 3D model may be due to the delayed diffusion of its components into the BSE “The Dermal Equivalent, ED” hydrogel, which makes it difficult for the extract to penetrate to the cells. The results of studies using Chitopran^®^ extract have shown its biocompatibility with DFs in 2D and 3D culture conditions. However, Chitopran^®^ has been found to have a strong cytotoxic effect on DFs when placed into direct contact with the BSE “The Dermal Equivalent, ED”. The cytotoxic effect of Chitopran^®^, when placed into direct contact with BSE may be due to the presence of acetic acid, which is a component of the molding solution used to form chitosan polymer fibers. The absence of a cytotoxic effect when using Chitopran^®^ extracts can be explained by the reduction in the concentration of acetic acid due to dilution during extraction. The results from studies with Granuflex^®^ extract have generally shown its biocompatibility with DFs in 3D and 2D models, although the findings in the 2D model are unclear and require further investigation (Table 4). The cytotoxic effect of Granuflex^®^ in direct contact with the BSE may be due to the sorbent properties of this WD. Due to the cytotoxicity shown by Chitopran^®^ and Granuflex^®^ on DFs in direct contact with BSE “The Dermal Equivalent, ED”, use of these WDs as a secondary WD for BSE “The Dermal Equivalent, ED” is not recommended. The use of Syspyr-derm^®^, Branolind^®^ N, Aquacel^TM^ adhesive foam, and Aquacel™Ag^+^ as a secondary WD for BSE “The Dermal Equivalent, ED” does not seem to be advisable, as they showed a strong cytotoxic effect on DFs inside CH in direct contact with the hydrogel BSE in vitro experimental conditions (Table 4) this may be directly related to the sorbing properties of these WDs. In addition, extracts based on these materials also had a pronounced cytotoxic effect on cells cultured under 2D conditions. The low biocompatibility of these materials with BSE “The Dermal Equivalent, ED” may be related to the delayed diffusion of cytotoxic components from the extracts into the hydrogel.

Thus, the screening revealed that out of the WDs selected for the study, only Parapran^®^ with chlorhexidine showed a high biocompatibility with the BSE “The Dermal Equivalent, ED” and can be used in combination with it as a secondary WD. However, it is not an optimal secondary WD for this BSE at the same time. It should be noted that the biodegradation of CH and the preservation of DFs function in the composition of the BSE require a moist environment, which Parapran^®^, as a mesh WD, cannot provide when used alone. In this regard, when using WD Parapran^®^ in combination with this BSE, the use of additional dressings such as sterile gauze wipes soaked in 0.9% saline sodium chloride solution, as well as additional fixation techniques, is necessary. Additionally, this type of bandage dries up within 4 h and requires periodic hydration throughout the day, which can be time-consuming and difficult for both patients and medical staff. Therefore, the question of using various WDs in combination with hydrogel BSE “The Dermal Equivalent, ED” is currently a debated issue that requires further research to find or develop an optimal alternative WD that would help fix the BSE onto the wound, protect it from drying, environmental factors including antiseptics, and damage from dressings, while also providing concomitant pharmacotherapy. One such approach is the development of a modified version of BSE “The Dermal Equivalent, ED”. Using chemical and physical crosslinking methods, collagen hydrogels can be engineered to exhibit a range of properties that make them adaptable and suitable for various applications when used as WD [20]. Currently, our research focuses on the development of a two-layered collagen BSE, where the lower layer consists of collagen hydrogel with human skin cells and the upper layer is a cell-free collagen hydrogel that has been modified with various crosslinking agents and antiseptics. Compared to single-layer hydrogels, multilayer hydrogels represent an important and novel branch of hydrogels [39]. Multilayer hydrogel BSE, with the upper layer serving as a protective barrier against external pathogens and maintaining a moist environment, and the lower layer acting as a carrier for cells, has the potential to meet the therapeutic requirements of various stages and aspects of the wound healing process.

## 3. Materials and Methods

### 3.1. Test Wound Dressings

The study focused on 11 different types of modern commercial wound dressings: Syspur-derm^®^, Parapran^®^, Lomatuell^®^ H, Voskopran^®^, Metalline^®^, Granuflex^®^, Chitopran^®^, HydroTac^®^ transparent, Branolind^®^ N, Aquacel^TM^ adhesive foam, Aquacel^TM^Ag^+^. (Table 2) and BSE “The Dermal Equivalent, ED” developed at the Institute of Cytology of the Russian Academy of Sciences.

These commercial WDs are included in the list of most used medical devices, according to the national clinical guidelines and the procedure for providing medical care according to the profile “surgery (combustiology)” [28,29].

**Figure 4:**
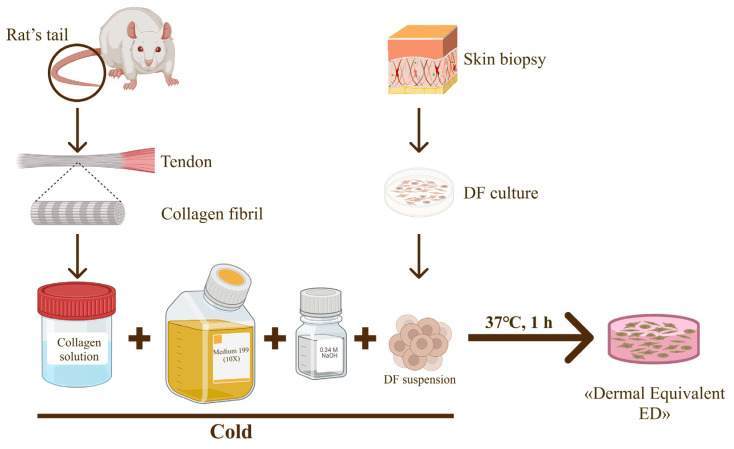
Schematic illustration of the preparation process BSE “The Dermal Equivalent, ED”.

Type I collagen obtained from rat tail tendons by acid extraction [40] and human dermal fibroblasts (DFs) were used to prepare hydrogel BSE “The Dermal Equivalent, ED”. To perform this, collagen solution was mixed with a concentrated (10×) medium 199 (Sigma-Aldrich, St. Louis, MO, USA), and the pH of the mixture was adjusted to neutral by adding 0.34 M NaOH (Sigma-Aldrich, St. Louis, MO, USA). Then a suspension of cells in complete nutrient medium DMEM/F-12 was added. The final concentration of DFs in the finished hydrogel BSE was 1.0 × 10^5^ cells/mL. The final collagen concentration in the hydrogel was 2 mg/mL. BSE preparation was conducted in cold conditions using a desktop cooling workstation, the Ice Free Cool Box (Nest, Wuxi, China). The collagen hydrogel containing cells, prepared using this method, was poured into 24-well and 48-well plates in volumes of 500 μL and 250 μL, respectively. The cells were then incubated in a CO_2_ incubator at 37 °C for one hour until complete polymerization. The scheme for the preparation of BSE “The Dermal Equivalent, ED”, is shown in Figure 4.

### 3.2. Cell Culture

As a model cellular test system (2D and 3D models), the study used immortalized human DFs isolated from eyelid skin biopsies obtained during cosmetic surgery, with the voluntary informed consent of patients who had negative results of a mandatory blood tests for markers of hepatitis B and C, HIV, and RW. The cells were cultured at 37 °C in a CO_2_ incubator, in an atmosphere with 5% CO_2_, using a DMEM/F12 nutrient medium (Gibco ™, Thermo Fisher Scientific, Waltham, MA, USA), supplemented with 10% fetal bovine serum (FBS) (HyClone, Logan, UT, USA), and 1% penicillin-streptomycin (Gibco™, Thermo Fisher Scientific, Waltham, MA, USA).

### 3.3. Design and Research Methods

The biocompatibility of various modern WDs was evaluated in vitro using their effect on the viability of human dermal fibroblasts (DFs) (2D model). To assess the effect of WDs on cultured cells, extracts obtained during incubation WD with culture media was used instead of the actual WD. The biocompatibility of WDs with the BSE “The Dermal Equivalent, ED” was investigated in vitro by examining the effect of WDs on the cellular component of the BSE (DFs) (3D model) using extracts and in direct contact the studied WDs with BSE “The Dermal Equivalent, ED”. All experimental procedures followed the International Organization for Standardization (ISO) 10993-5 [27]. The viability of DFs was assessed by their morphology, metabolic activity, using light microscopy and the MTT assay.

### 3.4. Extraction Method

The extraction method is based on preparing liquid extracts from a material under conditions similar to those used in clinical practice, considering the specific properties of the material. Extract preparation was performed according to the ISO 10993-5 standard [27]. To prepare WD’s extracts, a ratio of the surface area of the WD in square centimeters to the volume of the nutrient medium in milliliters was used, which was equal to 1:2. For this purpose, the WDs were cut into fragments and placed in complete DMEM/F12 medium and incubated at 37 °C for 3 days. Variants of extracts from these WDs were used in the research.

### 3.5. Determination of Cell Viability Under 2D Conditions

To assess the effect of WDs on the metabolic activity of human DFs under 2D cultivation, two sets of experiments were conducted using extracts obtained from WDs. Cells were seeded in 96-well plates at concentrations of 5000 and 1000 cells per well, in 200 μL of standard culture medium. For the first set of experiments, replacement of the standard culture medium with extract occurred immediately after cell adhesion. For the second set, it happened 7 days later (when cells had formed a monolayer). Cells were cultured in extracts at 37 °C in a humidified atmosphere of 5% CO_2_ for 3 days. Morphological changes in the cells were monitored during cultivation. DFs grown under standard conditions served as controls. After the end of cultivation, an MTT assay was performed according to the standard protocol [41]. Briefly, the extracts in the wells were replaced with 100 μL of a medium containing 0.5 mg/mL of MTT, and the cells were incubated for 4 h at 37 °C. After the incubation time, the medium was completely removed and replaced with 100 μL DMSO to extract the intracellular formazan crystals. The optical density of the final formazan solution in DMSO was measured using a Fluorofot «Charity analyzer» (Moscow, Russia) at a wavelength of 570 nanometers and a reference wavelength of 630 nanometers.

To analyze the effect of WDs on the structure of the actin cytoskeleton in DFs, cells were seeded on cover glasses placed in wells of a 24-well plate. After cell adhesion, the standard culture medium was replaced with WDs’ extracts. The cells were cultured in extracts at 37 °C in a CO_2_ incubator with 5% CO_2_ atmosphere for 3 days. At the end of the incubation period, the cells were fixed with a 4% paraformaldehyde solution and permeabilized with 0.1% Triton X-100. Cells were then stained with the Rhodamine Phalloidin fluorescent dye (Invitrogen™, Thermo Fisher Scientific, Waltham, MA, USA) and enclosed in a medium containing DAPI (Sigma-Aldrich, St. Louis, MO, USA). The analysis of the actin cytoskeleton was carried out using a laser scanning confocal microscope (LSCM), Olympus FLUOVIEW FV3000 (Olympus Corporation, Tokyo, Japan). The images were processed using Fiji software (ImageJ 2.3.0; Java 1.8.0_322). 

### 3.6. Determination of Cell Viability Under 3D Conditions

The effect of WDs on the metabolic activity of human DFs cultured inside a collagen hydrogel, as part of a BSE “The Dermal Equivalent, ED”, was evaluated using two methods: the extraction method and the direct contact method (Figure 3A).

Unpolymerized collagen hydrogel with a cellular suspension was placed in wells of 24- and 48-well plates in a volume of 500 μL and 250 μL, respectively. After the collagen hydrogel had completely polymerized, culture medium was added to the wells and the BSE was incubated in a CO_2_ incubator at 37 °C for 2 days. After this time, the standard culture medium was removed from the wells of the 48-well plate and replaced with extracts. Also, WDs fragments of equal surface area were placed on the BSE surface in the wells of the 24-well plate after removing the standard culture medium. Incubation of the BSE with extracts and WD fragments was performed at 37 °C in a CO_2_ incubator in an atmosphere containing 5% CO_2_ for 3 days. During the incubation period, the morphological state of cells inside the BSE was assessed. The control group was the BSE with DFs incubated under standard conditions. At the end of the incubation period, an MTT assay was performed, adapted by us to assess the viability of the cells cultured inside the hydrogel. At the end of the incubation period, the extracts were removed from the wells of the 48-well plate and the WDs fragments were removed from the 24-well plate. A medium containing MTT (Sigma-Aldrich, St. Louis, MO, USA) at a concentration of 0.5 mg/mL was then added to both plates. The plates were placed in a thermoshaker orbital shaker for 5 h at 37 °C and a speed of 240 rpm. After the incubation period, the MTT solution was replaced with an equal volume of dimethyl sulfoxide (DMSO) solution. The intracellular formazan crystals were then extracted by shaking the plates continuously for 24 h at room temperature. After that, 100 µL of the contents from each well were transferred to a 96-well plate for measuring the optical density of the solutions using a spectrophotometer, following a standard protocol.

### 3.7. Statistical Analysis

Statistical analysis of the data and plotting were performed using GraphPad Prism (version 8). Differences between results were analyzed using the one-way ANOVA followed by Dunnett’s multiple comparisons test for significance. For all statistical comparisons, * *p* < 0.05, ** *p* < 0.01, *** *p* < 0.001and **** *p* < 0.0001 were considered significant. The results are presented as mean ± standard deviation.

## 4. Conclusions

Various strategic approaches and methods are used to treat skin wounds. Most of these methods are based on using wound dressings (WDs) to protect wounds from further damage and infection and to accelerate healing. To date, many different dressings have been developed for various treatment protocols. New modern WDs are also emerging, developed by different manufacturers in various forms and with different characteristics. Among these, bioengineered skin equivalents (BSEs) occupy a special place. At the same time, with such a wide variety of WDs available, it is difficult to fully understand the advantages of each one individually, as well as the potential for combining them to optimize wound healing. The heterogeneity of these WDs and lack of standardized protocols may limit their clinical utility or make them ineffective. Our in vitro screening of the biocompatibility of 11 modern WDs of various types used in the treatment of acute and chronic wounds has demonstrated that only 5 of them do not negatively affect the morphofunctional state of human DFs and therefore have high biocompatibility. And only one of them, Parapran^®^ with chlorhexidine, has shown high biocompatibility with the BSE “The Dermal Equivalent, ED” and can be used in combination with it as a secondary WD; however, this dressing is not optimal for this purpose due to its properties. We believe that a solution to this problem could be the development of a new interactive WD with high biocompatibility to skin cells and the ability to solve the methodological challenges of clinical application of the hydrogel BSE “The Dermal Equivalent, ED”.

## Figures and Tables

**Table 1:** Summary of experimental parameters (2D model) of MTT assay.

In Vitro Biocompatibility WDs								
High			Requires Clarification			Low		
WD	M1, %	M2, %	WD	M1, %	M2, %	WD	M1, %	M2, %
Parapran^®^ (with chlorhexidine)	116.0 ± 5.7	114.1 ± 4.0	Granuflex^®^	98.6 ± 3.5	80.4 ± 3.5	Syspyr-derm^®^	32.7 ± 0.5	42.5 ± 1.7
Lomatuell^®^ H	112.1 ± 5.6	106.6 ± 5.4				HydroTac^®^ transparent	32.8 ± 1.5	43.2 ± 0.9
Voskopran^®^	100.7 ± 2.7	99.1 ± 2.7				Branolind^®^ N	32.9 ± 0.8	36.7 ± 0.8
Metalline^®^	110.8 ± 3.4	106.9 ± 3.3				Aquacel^TM^ adhesive foam	73.9 ± 3.4	69.0 ± 3.9
Chitopran^®^	95.6 ± 4.6	102.4 ± 4.2				Aquacel^TM^ Ag^+^	32.9 ± 0.5	38.4 ± 1.7

M1—the viability of DFs cultured in extracts added immediately after adhesion (Mean ± SD). M2—the viability of DFs cultured in extracts added 7 days after adhesion (Mean ± SD).

**Table 2:** Description of wound dressings.

		WD’s Type	Description	Main Characteristics	Application	Advantages	Disadvantages
1	Syspur-derm^® 1^	Hydro-cellular dressings	Double-layer polyurethane sponge material	The wound dressing promotes intensive and effective cleaning of the infected wound surface thanks to the adhesion of wound discharge. The inner layer of the sponge serves as a matrix for the formation of new cell tissues during the formation of granulation tissue in clean wounds. The outer film helps reduce drying of the wound and acts as an external barrier against infection. The outer layer also has a high degree of oxygen permeability to maintain temperature parameters.	Cleansing of wounds of thermal and mechanical origin, stimulation of healing, and formation of new granulation tissue in chronic or acute wounds with a slow recovery process, such as bedsores, radiation injuries, diabetic ulcers, and trophic changes.	Effective and fast wound cleansing thanks to the sorption capacity of the product. Acceleration of the reparative process in tissues and stimulation of granulation tissue formation. Painlessness and pleasant aroma during bandaging. Protection of the wound from secondary infection and drying out. Possibility of modeling the bandage in different planes during wound closure. No maceration or allergic reaction around the skin.	Monitoring of the wound dressing should be performed daily. If any fluid accumulation or signs of infection are noted, the dressing should be changed. To ensure complete cleaning of the wound, it is recommended to replace the compress every 12–24 h.
2	Parapran^® 2^		Knitted mesh made of 100% cotton, paraffin, chlorhexidine bigluconate	Antimicrobial atraumatic dressing with chlorhexidine is a local therapeutic and prophylactic antiseptic that is applied to wounds. The dressing contains chlorhexidine in a paraffin composition that gradually releases the antiseptic over time, providing a long-lasting antimicrobial effect on the wound.	Purulent and purulent-necrotic wounds of various etiologies: burns, bedsores, trophic ulcers, frostbite, purulent-inflammatory diseases of soft tissues, abscesses, and phlegmon.	Atraumatic and painless bandaging. Effective prevention of secondary infections. Creation of a humid environment. The ability to adjust the bandage in different directions.	Individual intolerance to the constituent components.
3	Lomatuell^®^ H ^3^	Atraumatic	Cotton bandage with paraffin impregnation	A sterile, coarse-mesh cotton bandage that is impregnated with a neutral, water-repellent (hydrophobic) ointment. The mesh structure allows for free absorption of any secretions from the wound and directs them to a secondary, absorbable bandage.	First and second degree burns, superficial wounds, abrasions, cracks, radiation-induced wounds, trophic ulcers on the shin, and donor and recipient sites during transplantation.	The wound dressing provides good ventilation for the wound and has analgesic, antibacterial, and wound healing effects. It can be used in conjunction with any antibiotics or antiseptic medications.	Daily dressings. Individual intolerance to the constituent components.
4	Voskopran^® 2^	Anti-inflammatory ointment dressing	Knitted polyester mesh, beeswax, propolis, vitamin E, Levomekol ointment (dioxomethyltetrahydropyrimidine, chloramphenicol)	A combined drug that contains the antibiotic chloramphenicol and the immunostimulating agent methyluracil. This medication provides an anti-inflammatory and anti-microbial effect on the wound. Due to its polyethylene glycol base, “Voskopran” ointment has a long-lasting absorption effect.	Wounds with signs of infection, including purulent wounds and trophic ulcers. Bedsores, diabetic foot ulcers, and wounds from prolonged pressure “crash” syndrome. Gangrene, purulent inflammatory skin diseases, such as boils and phlegmons. Burns of I-III degrees, lacerations, and animal and insect bites. Gunshot wounds and dermatitis.	High antimicrobial activity not only against aerobic Gram-positive and Gram-negative bacteria, but also against anaerobic microorganisms. Long-lasting sorption effect. Optimization of the processes of wound repair.	Individual intolerance to the constituent components.
5	Metalline^® 3^	Sorbing	The wound layer is a non-woven viscose material coated with aluminum. The absorbent layer is non–woven fabric. The outer layer is a thin, breathable non-woven fabric	It consists of three layers. The outer layer is made of a non-woven fabric. The middle layer is made from a fibrous, absorbent non-woven material. And the inner layer, which is in contact with the wound, is made from thin, perforated aluminum foil. This ensures the atraumatic properties of the medication.	This product is intended for use in various medical fields, including traumatology, surgery, dermatology, and phlebology. It is especially suitable for the treatment of extensive wounds, abrasions, and burns. It can also be used as a first aid bandage and for securing drains and tracheostomy tubes.	It does not stick to the wound surface. A painless bandage change. Gentle wound care. Air and vapor permeability. Soft and plastic structure. Optimal drainage capacity. It is waterproof, (the patient can take a bath or shower with a bandage applied.)	Individual intolerance to the constituent components.
6	Granuflex^® 4^	Hydrocolloid	A hydrocolloid dressing consists of an inner layer that is in contact with the wound (gelatin, pectin, sodium carboxymethylcellulose) and an outer layer (polyurethane film)	A wound dressing consists of an inner layer in contact with the wound, which is made of hydrocolloids, an intermediate layer of polyurethane foam, and an outer layer made of polyurethane film. The wound dressing interacts with wound exudate to create a moist environment, which supports the healing process. It also absorbs exudate and promotes autolytic cleansing of the wound.	Wound dressing is used to treat chronic, exudating wounds such as leg ulcers, pressure sores, minor burns, donor sites (after hemostasis has been achieved) and other types of wounds that are granulating. When applied to wounds with dry, peeling, or necrotic tissue, the dressing helps prevent the loss of moisture from the skin’s surface. This helps rehydrate dead tissue, which can then be removed by autolysis.	Adhesive foam with a thin layer of hydrocolloid extends beyond the central hydrocolloid mass, creating a border with low-profile edges for added safety in hard-to-reach areas.	Individual intolerance to the constituent components. It is important to carefully monitor the condition of the wound and all treatment should be performed under the supervision of a doctor. The use of Granuflex in the presence of an anaerobic infection is not recommended. Improper use or frequent changes in bandages can lead to skin irritation or peeling.
7	Chitopran^® 5^	Bioplastic coating	Biopolymer dressing based on chitosan nanofibers in the form of a non-woven fabric	It is a bioplastic material composed of randomly oriented chitosan fibers with a diameter between 300 and 400 nanometers. In the treatment of patients, wound coverings measuring 10 × 10 cm were used.	Local treatment of superficial granulating, slow-healing, long-lasting wounds in the regenerative stage, I–III degree burns, trophic ulcers, pressure sores, and frostbite. Temporary closure of III degree burn wounds to prepare them for autografting, closure of donor sites. Healing of oral wound surfaces.	It provides the necessary level of moisture, air circulation, and painless removal. It can be easily removed from the wound or absorbed by itself. It can also be used as a gauze, as the sterile Chitopran wound healing bandage (5 × 7.5 cm) is easy to shape. Suitable for sensitive, delicate skin.	Individual intolerance to the constituent components.
8	HydroTac ^®^ transparent ^1^	Hydrogel	A hydrogel dressing made of a polymer based on a hybrid of polyurethane and polyurea containing propylene glycol with a water content of about 60%.	Due to its high water content of about 60%, the HydroTac transparent bandage instantly creates and maintains a moist wound environment. This helps to rapidly reject necrotic masses and stimulates the processes of granulation and epithelialization.	The bandage is suitable for treating weakly exudating and dry, chronic wounds of various origins and any location, without clinical signs of infection, such as trophic venous ulcers, diabetic ulcers, pressure sores, and other poorly healing wounds, at the stages of granulation and epithelialization. It can also be used to treat grade II burns and donor sites.	A hydrogel dressing that can be used to treat dry, chronic, and poorly healing wounds, as well as wounds with low levels of exudate during the epithelialization phase. This dressing helps create and maintain an optimal moist wound environment, providing long-term hydration. It actively stimulates epithelialization and has a firm fit to the wound. The dressing can stay on the wound for up to seven days without the need for bandage changes.	HydroTac transparent dressing should not be used if the patient has a hypersensitivity to any of its ingredients. It should also not be used on clinically infected wounds or third-degree burns.
9	Branolind^®^ N ^1^		Coarse cotton fabric impregnated with an ointment composition: Peruvian balm (benzoic and cinnamic acid, vanillin, etc.), white petroleum jelly, cetomacrogol, glycerin monostearate, hydrogenated fat, medium-chain triglycerides.	The bandage has a soft and flexible structure, which allows it to tightly fit around the damaged area without causing pain or discomfort. The Peruvian balm in the dressing contains strong anti-inflammatory properties and helps accelerate the process of tissue regeneration. The Branolind N ointment with Peruvian balsam dressing provides reliable protection for wounds from infection and improves blood microcirculation. The bandage is made from coarse-mesh cotton fabric, which has a high degree of air permeability and secret permeability.	Abrasions, wounds, ulcers, scuffs and other superficial skin lesions; burns within the skin; donor wounds during skin transplantation; venous trophic ulcers; wounds in the treatment of diabetic foot; wounds with a high risk of scarring contractures.	Atraumatic and painless during bandages. Protection of the wound from secondary infection. Prevention of wound drying out. Possibility of adjusting bandages in different directions when closing wounds.	It is possible to develop dermatitis, contact sensitivity, and photosensitization as well as allergic reactions from Peruvian balsam. It is necessary to avoid contact of the product with the eyes, mucous membranes, and serous membranes.
10	Aquacel^TM^ adhesive foam ^4^		The non-woven layer that comes into contact with the wound surface is made of sodium carboxymethylcellulose. On top of that, there is a layer of polyurethane foam.	It comes into direct contact with the wound and absorbs a large amount of wound exudate and bacteria. It forms a soft gel, which creates the effect of micro-contouring the wound surface, creating and maintaining a moist environment for faster healing. The foam layer absorbs and retains a large amount of wound content. The outer film provides a barrier against viruses and bacteria and is waterproof, protecting the wound from external contamination. The silicone adhesive edge promotes a strong, skin-friendly adhesion, promoting an atraumatic change in the wound coating. Silver has a bactericidal effect on a wide range of bacteria, including antibiotic-resistant hospital strains.	Ulcers of the lower extremities (trophic, diabetic), infected wounds and wounds with an increased risk of infection, pressure sores with partial or complete skin damage to the entire depth, and post-traumatic wounds such as lacerations and abrasions.	The wound dressing absorbs a large amount of wound discharge, including bacteria. It helps remove non-viable tissue from the wound and cleans it, promoting faster healing. Silicone technology provides less trauma to the skin around the wound, and the dressing can remain in place for up to 7 days unless there are clinical indications for removing it.	Individual intolerance to the constituent components.
11	Aquacel^TM^ Ag^+ 4^		A non-woven bandage consists of two layers of sodium carboxymethylcellulose, with 1.2% silver and ethylenediaminetetraacetic acid. It also contains benzetonium chloride and is stitched together with a thread made from regenerated cellulose.	It contains EDTA and benzethonium chloride, thanks to which it is able to remove formed bacterial biofilms and prevent their formation, as well as increase the effectiveness of silver delivery to microorganisms.	Infected wounds or wounds with a high risk of infection, such as trophic ulcers in the lower extremities due to venous insufficiency or ischemia, diabetic foot ulcers, bedsores with partial or complete damage to tissue layers, surgical wounds, traumatic wounds, bleeding wounds after mechanical or surgical treatment, exudating wounds from cancerous skin tumors like fungoid tumors, granulomatous carcinoma, skin metastases, and Kaposi’s sarcoma. Wounds where the presence of bacteria may be a suspected cause of chronic non-healing.	Reduces the severity of pain syndrome when the bandage is applied to the wound; absorbs and retains excess fluid and toxic substances (e.g., bacteria) within the fibers of the bandage, thus reducing the risk of skin maceration and damage around the wound; precisely replicates the features of wound relief, reducing the dead space in the wound (in contact with the therapeutic bandage); and provides controlled release of silver ions as exudate accumulates on the bandage, with rapidly developing and stable antibacterial activity.	Allergic reaction to silver or sodium carboxymethylcellulose.

Manufacturers of wound dressings: ^1^—“Hartmann Group” (Germany) [31]; ^2^—“Biotechfarm” (Russia) [32]; ^3^—“Lohmann & Rauscher” (Germany) [33]; ^4^—“ConvaTec” (USA) [34]; ^5^—“Napoly” (Russia) [35].

**Table 3:** Summary of experimental parameters (3D model) MTT assay.

Biocompatibility WDs with BSE “The Dermal Equivalent, ED” in Direct Contact In Vitro		
WD	M1, %	M2, %
Parapran^®^	125.0 ± 6.0	97.4 ± 1.0
Metalline^®^	127.5 ± 5.6	88.9 ± 0.5
Lomatuell^®^H	117.7 ± 5.7	87.0 ± 1.0
HydroTac^®^ transparent	98.4 ± 2.6	86.8 ± 0.3
Voskopran^®^	117.3 ± 1.9	84.2 ± 4.9
Chitopran^®^	99.2 ± 9.8	75.9 ± 1.2
Granuflex^®^	105.0 ± 4.9	74.8 ± 0.7
Branolind^®^N	91.5 ± 1.3	63.4 ± 0.6
Syspur-derm^®^	97.2 ± 2.3	62.1 ± 2.3
Aquacel^TM^ adhesive foam	90.7 ± 3.2	56.9 ± 0.8
Aquacel^TM^Ag^+^	97.5 ± 2.8	55.4 ± 1.2

M1—the viability of DFs cultured in WDs’ extracts (Mean ± SD). M2—the viability of DFs cultured in contact with WD (Mean ± SD).

**Table 4:** Summary of experimental parameters MTT assay.

Biocompatibility WDs In Vitro				
WD	M1, %	M2, %	M3, %	M4, %
Parapran^®^	116.0 ± 5.7	114.1 ± 4.0	125.0 ± 6.0	97.4 ± 1.0
Metalline^®^	110.8 ± 3.4	106.9 ± 3.3	127.5 ± 5.6	88.9 ± 0.5
Lomatuell^®^H	112.1 ± 5.6	106.6 ± 5.4	117.7 ± 5.7	87.0 ± 1.0
HydroTac^®^ transparent	32.8 ± 1.5	43.2 ± 0.9	98.4 ± 2.6	86.8 ± 0.3
Voskopran^®^	100.7 ± 2.7	99.1 ± 2.7	117.3 ± 1.9	84.2 ± 4.9
Chitopran^®^	95.6 ± 4.6	102.4 ± 4.2	99.2 ± 9.8	75.9 ± 1.2
Granuflex^®^	98.6 ± 3.5	80.4 ± 3.5	105.0 ± 4.9	74.8 ± 0.7
Branolind^®^N	32.9 ± 0.8	36.7 ± 0.8	91.5 ± 1.3	63.4 ± 0.6
Syspur-derm^®^	32.7 ± 0.5	42.5 ± 1.7	97.2 ± 2.3	62.1 ± 2.3
Aquacel^TM^ adhesive foam	73.9 ± 3.4	69.0 ± 3.9	90.7 ± 3.2	56.9 ± 0.8
Aquacel^TM^Ag^+^	32.9 ± 0.5	38.4 ± 1.7	97.5 ± 2.8	55.4 ± 1.2

M1—the viability of DFs cultured in extracts added immediately after adhesion. Two-dimensional Model (Mean ± SD). M2—the viability of DFs cultured in extracts added 7 days after adhesion. Two-dimensional Model (Mean ± SD). M3—the viability of DFs cultured in WDs’ extracts. Three-dimensional Model (Mean ± SD). M4—the viability of DFs cultured in contact with WDs. Three-dimensional Model (Mean ± SD).

## Data Availability

The original contributions presented in this study are included in the article. Further inquiries can be directed to the corresponding author.
